# Supplementary material for: The desmoplakin tail domain position in the desmosomal plaque is isoform dependent
Source: J Cell Sci. 2025 Sep 8;138(17):jcs263906. doi: 10.1242/jcs.263906 (PMC12450459; doi:10.1242/jcs.263906)
Supplement: Supplementary information [file joces-138-263906-s1.pdf]

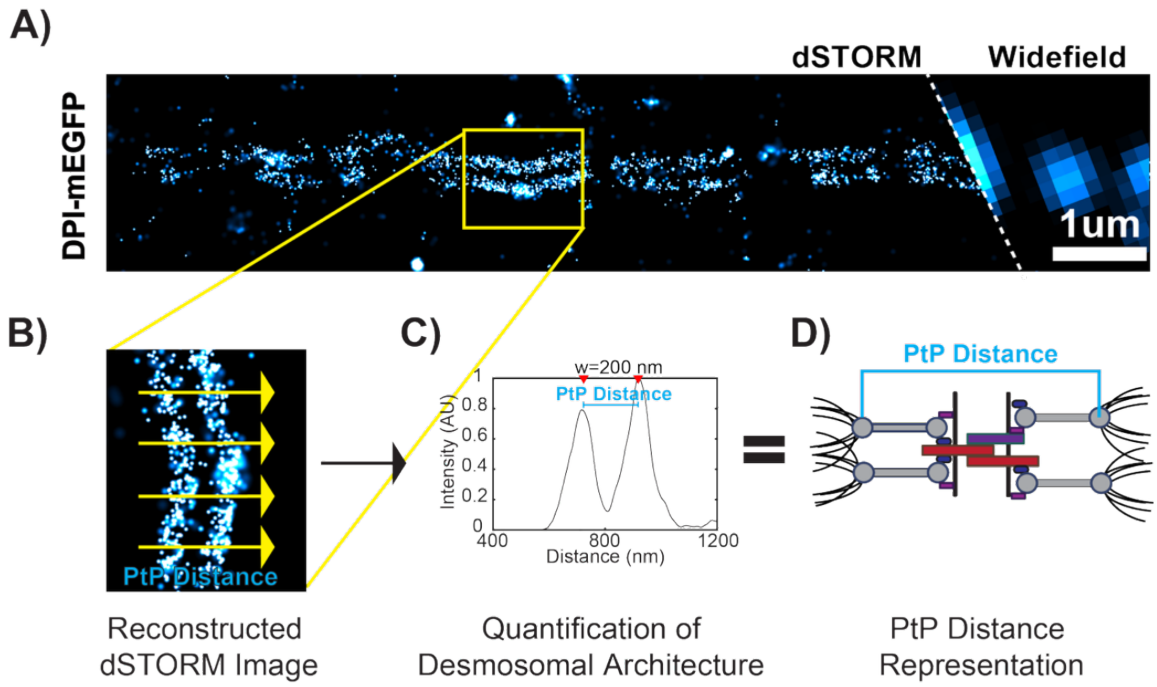

Fig. S1. dSTORM Analysis Pipeline: **A)** Overlay of widefield and dSTORM reconstructed image of DPI-mEGFP HaCaT Cells. **B)** Cropped reconstructed desmosome with yellow arrows indicating the linescan used to measure the plaque-to-plaque (PtP) distance. **C)** dSTORM analysis output graph of intensity as a function of distance along the linescan. The distance between the two peaks is the PtP distance. **D)** Schematic representation of PtP distance.

Journal of Cell Science • Supplementary information

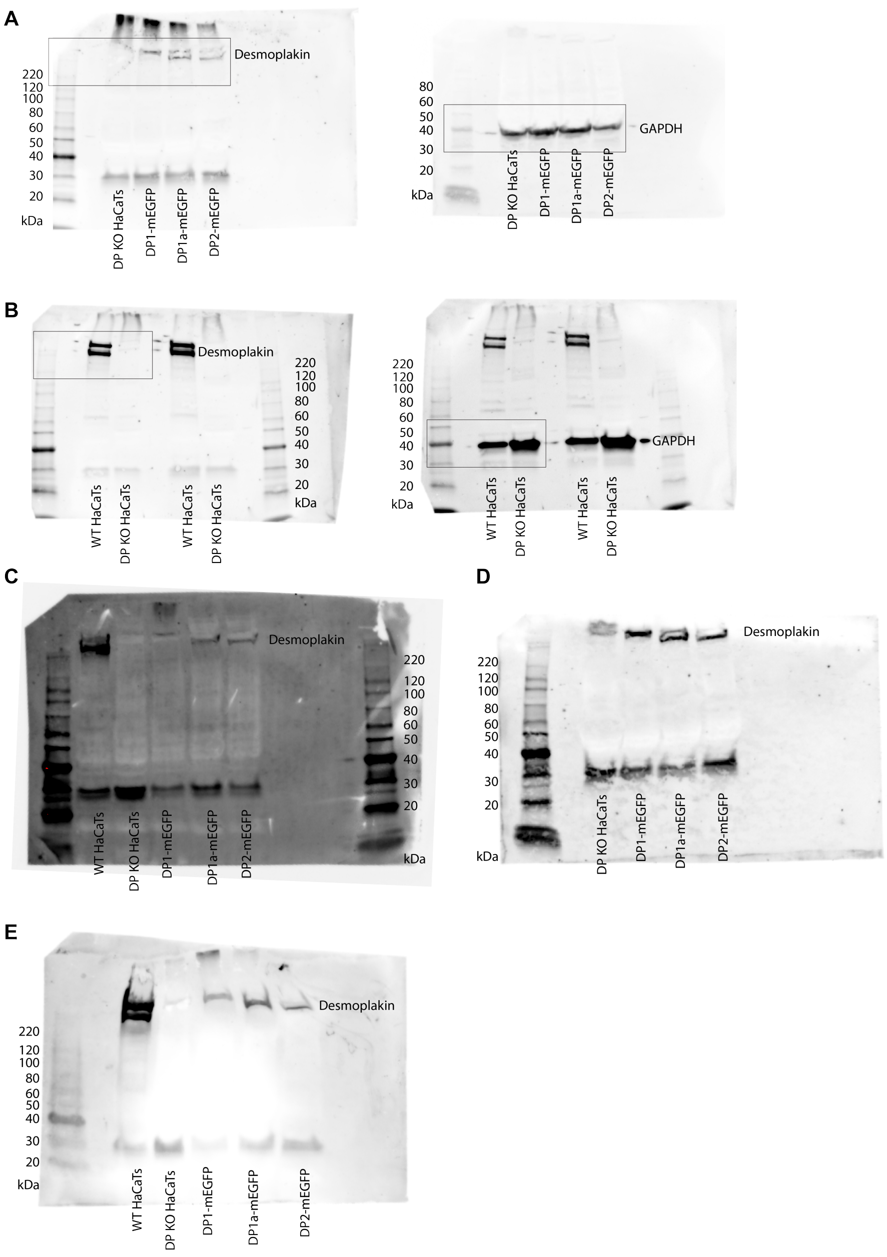

Fig. 2. Raw Transparency Blots of Desmoplakin and GAPDH. A) Immunoblot of DP (left) and GAPDH (right) from DP-mEGFP HaCaT and DP KO HaCaT lysates. **B)** Immunoblot probing for DP (left) and GAPDH (right) from WT and DP KO HaCaT lysates **C)** Immunoblot for DP from DP-mEGFP HaCaT DP KO and WT HaCaT lysates. **D)** Immunoblot for DP from DP-mEGFP HaCaT DP KO lysates. **E)** Immunoblot of DP from DP-mEGFP HaCaT DP KO and WT HaCaT lysates. A total of 5 western blots are presented. 4 blots show DP-mEGFP isoforms (A, C, D, E) and 3 blots show WT and KO HaCaTs (B, C, E). Predicted molecular weights: DPI-mEGFP 359 kDa, DPIa-mEGFP 306 kDa, DPII-mEGFP 287 kDa, DPI 332 kDa, DPII 260 kDa, and GAPDH 37 kDa. All blots use antibodies indicated in Materials and Methods.
